# *Ginkgo biloba* attenuated detrimental inflammatory and oxidative events due to *Trypanosoma brucei rhodesiense* in mice treated with melarsoprol

**DOI:** 10.1371/journal.pntd.0012103

**Published:** 2024-04-15

**Authors:** Janet Khatenje Wendo, James Mucunu Mbaria, James Nyabuga Nyariki, Alfred Orina Isaac

**Affiliations:** 1 The University of Nairobi, Department of Public Health, Pharmacology and Toxicology, Kangemi (Nairobi), Kenya; 2 The Technical University of Kenya, Department of Pharmaceutical Sciences and Technology, Nairobi, Kenya; 3 The Technical University of Kenya, Department of Biochemistry and Biotechnology, Nairobi, Kenya; Universidade Federal de Minas Gerais, BRAZIL

## Abstract

**Background:**

The severe late stage Human African Trypanosomiasis (HAT) caused by *Trypanosoma brucei rhodesiense* (T.b.r) is characterized by damage to the blood brain barrier, severe brain inflammation, oxidative stress and organ damage. Melarsoprol (MelB) is currently the only treatment available for this disease. MelB use is limited by its lethal neurotoxicity due to post-treatment reactive encephalopathy. This study sought to assess the potential of *Ginkgo biloba* (GB), a potent anti-inflammatory and antioxidant, to protect the integrity of the blood brain barrier and ameliorate detrimental inflammatory and oxidative events due to T.b.r in mice treated with MelB.

**Methodology:**

Group one constituted the control; group two was infected with T.b.r; group three was infected with T.b.r and treated with 2.2 mg/kg melarsoprol for 10 days; group four was infected with T.b.r and administered with GB 80 mg/kg for 30 days; group five was given GB 80mg/kg for two weeks before infection with T.b.r, and continued thereafter and group six was infected with T.b.r, administered with GB and treated with MelB.

**Results:**

Co-administration of MelB and GB improved the survival rate of infected mice. When administered separately, MelB and GB protected the integrity of the blood brain barrier and improved neurological function in infected mice. Furthermore, the administration of MelB and GB prevented T.b.r-induced microcytic hypochromic anaemia and thrombocytopenia, as well as T.b.r-driven downregulation of total WBCs. Glutathione analysis showed that co-administration of MelB and GB prevented T.b.r-induced oxidative stress in the brain, spleen, heart and lungs. Notably, GB averted peroxidation and oxidant damage by ameliorating T.b.r and MelB-driven elevation of malondialdehyde (MDA) in the brain, kidney and liver. In fact, the co-administered group for the liver, registered the lowest MDA levels for infected mice. T.b.r-driven elevation of serum TNF-α, IFN-γ, uric acid and urea was abrogated by MelB and GB. Co-administration of MelB and GB was most effective in stabilizing TNFα levels. GB attenuated T.b.r and MelB-driven up-regulation of nitrite.

**Conclusion:**

Utilization of GB as an adjuvant therapy may ameliorate detrimental effects caused by T.b.r infection and MelB toxicity during late stage HAT.

## Author summary

Human African Trypanosomiasis (HAT) is a lethal disease caused by *Trypanosoma brucei gambiense* or *Trypanosoma brucei rhodesiens*e (T.b.r) protozoan parasites. The only treatment available for the late, CNS stage due to T.b.r, is melarsoprol (MelB). Notably, MelB has an arsenic moiety, that makes it very neurotoxic, causing fatal post-treatment reactive encephalopathy in about 5% of those treated. This level of drug-induced mortality is unacceptable in this day and age. The question is, how can we block T.b.r-induced organ damage and safely administer MelB? In the late stage HAT, parasites wreak havoc by inducing severe oxidative stress, inflammation and breach of the blood brain barrier (bbb); while on the other hand, MelB is driving lethal encephalopathy. We used a HAT mouse model and sought to elucidate the potential of a standardized GB extract–a potent antioxidant anti-inflammatory, to alleviate T.b.r-driven damage to the bbb, induction of oxidative stress and inflammation; while ameliorating MelB-driven neurotoxic effects. According to the results, GB protected from lethal parasite induced organ damage, as well as MelB toxicity, providing a novel approach for development of an adjuvant therapy.

## Introduction

Human African Trypanosomiasis (HAT) is a neglected disease affecting 36 countries in sub-Saharan Africa [1]. Sixty million people are at risk of infection and approximately 300,000 people are infected annually [2]. Generally, the disease is fatal if left untreated or if the treatment is inadequate. Consequently, HAT is considered a crucial public health concern in Africa [3].

It has been noted that the disease burden among individuals affected is usually high (Shaw et al [4]. The Disability Adjusted Life Years, or DALY (an estimate of the number of years of life lost due to premature death and disability) for every premature death is between 25 and 33 years. The burden remains high on both livelihoods and households even for those patients who have been successfully treated. The estimated burden ranges from 1.5 to 10 months of earnings. This is despite the fact that the diagnosis and treatment of the disease is freely given. The Food and Agriculture Organization estimates US$ 1.5 billion is lost annually due to HAT [5].

Transmission of the disease occurs when an infected tsetse fly of the *Glossina* species, bites a person [6]. Protozoan parasites namely *Trypanosoma brucei gambiense* and *Trypanosoma brucei rhodesiense* (T.b.r) are known to cause infection. The former leads to persistent disease in Central and West Africa whereas the latter causes severe disease in Eastern and Southern parts of Africa [7]. The early stage of HAT, also referred to as the haemo-lymphatic stage is marked by the growth and proliferation of the trypanosomes in subcutaneous tissues, blood and lymph. The late stage i.e. neurological or meningo-encephalitic stage presents with invasion of the CNS by the trypanosomes after crossing the blood-brain barrier. The severe encephalitic phase of HAT caused by T.b.r leads to neuronal inflammation which is distinguished by enormous intrusion of effector inflammatory cells, swelling of the parenchymal vessels accompanied by encephalitis [8].

Various studies suggest that damage to the blood brain barrier is a key feature that worsens the extent of inflammation in the CNS during severe late-stage disease [8]. According to Sternberg et al [9], CNS inflammation is characterised by elevated levels of pro-inflammatory cytokines IFN-γ and TNF-α, accompanied by acute neuro-inflammatory reactions. On the other hand, anti-inflammatory cytokine IL-10 in the brain plays a protective role. As a result, IL-10 is a key immunomodulator in trypanosomiasis disease. Furthermore, activated macrophages bring about cytokines IFN-γ, IL-1, IL-6 and TNF-α, which cause acute phase reactions and also help the parasite to grow [10].

Melarsoprol (MelB) is currently the only drug available for treating late-stage HAT due to T.b.r, because of its ability to cross the blood-brain barrier. This is despite the fact that it causes extreme toxicities with 10% of patients on melarsoprol developing post-treatment reactive encephalopathy (PTRE). Unfortunately, about 5% of these patients succumb to this complication [11]. This is a very high drug-related mortality that can be avoided by the development of new safer drugs. Moreover, possibly, changes in the structure of MelB to make the drug less toxic are potential avenues for research. Other potential avenues for intervention include adjunct therapy that assuages MelB toxicity while improving treatment outcomes. Neurological seizures have resulted when melarsoprol is administered in the absence of active trypanosomes [12]. On this basis, reactive encephalopathy is mainly linked to melarsoprol. PTRE manifests in the brain as perivascular cuffings along with acute astrocytosis and persistent activation of the microglia [7]. Notably, this drug generates reactive oxygen species (ROS) together with similar radicals that change how the innate defense mechanisms of the body react to these reactive moieties [6]. In addition, melarsen oxide, which is a metabolite of melarsoprol, can bind to essential metabolic enzymes, generating a destructive vicious cycle of oxidative stress and cell damage [13]. These toxicities complicate the treatment of late stage HAT due to T.b.r. The need for a safe treatment for late-stage HAT due to T.b.r is of essence.

Control of severe oxidative/inflammatory damage induced by parasites in HAT is vital. Moreover, considering the significant inflammatory responses associated with PTRE and antioxidant system failure due to melarsoprol treatment, it is logical to use compounds, which have anti-inflammatory and antioxidant properties in HAT management. Previous studies have given beneficial results when compounds with potent antioxidant and anti-inflammatory properties are used alongside melarsoprol like CoQ10 and anthocyanins [6,14,15].

This study utilized the research grade standardised *Ginkgo biloba* (EGb 761 Tanakan tablets) as an adjunct therapy to melarsoprol. This product is obtained from leaves of *Ginkgo biloba Linne* tree. Generally, the curative outcome is believed to be due to numerous active components in GB as opposed to any single one of them. The mixture of biologically active ingredients present in GB has pleiotropic physiological effects, including the inhibition of amyloid-formation [16–17], antioxidant activity [18–19], anti-apoptosis activity [20], vasodilatory effect and inhibition of platelet aggregation [21–22]. Other actions are anti-inflammatory, immunomodulation, neuroprotection and DNA repair [23]. Notably, GB has demonstrated beneficial effects in some of the pathologies similar to those triggered by melarsoprol toxicity and CNS HAT such as cognitive impairment, vascular-driven dementia, oxidative stress and inflammation [23]. These prior findings were compelling, making it rational to use GB in this study, given that vascular dysfunction, inflammation and oxidative stress are vital pathological events both in late stage HAT and in toxicities driven by melarsoprol therapy. This study assessed the potential of orally administered *Ginkgo biloba* in ameliorating negative physiological and biochemical events caused by *Trypanosoma brucei rhodesiense* in mice treated with melarsoprol.

## Materials and methods

### Ethical statement

The present study used male Swiss white mice and the experimental procedures were carried out by adhering to the 3R rules and the ARRIVE checklist which is critical for reporting animal research [24]. Animal handling, training and all the necessary experimental techniques and protocols involving mice in the current study were sought and approved by Faculty of Veterinary Medicine, University of Nairobi Committee on Biosafety and Animal Care and Use (FVM BAUEC/2022/351). Consequently, all the necessary experiments were performed in compliance with the recommendations of the Helsinki declaration on guiding principles on care and use of animals. Humane endpoints were monitored to reduce any observable suffering.

### Experimental design

Male Swiss white mice were randomly assigned into six experimental groups (n = 10 per group). Group one was the normal control group; group two was infected with 5.0 × 10^4^ trypanosome; group three was infected with 5.0 × 10^4^ trypanosomes and after turning positive, intraperitoneally treated with melarsoprol once daily for ten days at a dose of 2.2 mg/kg bwt; group four was infected with 5.0 × 10^4^ trypanosomes and administered orally with *Ginkgo biloba* extract (GB) once daily at a dose of 80 mg/kg bwt after turning positive for 30 days, which was the last day of treatment; group five was given GB once daily from the beginning of the experiment for 14 days at a dose of 80 mg/kg bwt before infection with 5.0 × 10^4^ trypanosome. The pre-treatment was meant to help establish adequate serum and tissues levels of GB components, and consequently result in better protection from T.b.r and MelB toxicity. Addition of GB before infection will help determine if prior intake of GB can be more effective than administration post infection with T.b.r. Group six was infected with 5.0 × 10^4^, given GB once daily for 14 days at a dose of 80 mg/kg bwt, then concurrently with intraperitoneal melarsoprol at a dose of 2.2 mg/kg bwt for 10 days, and continued with GB at a dose of 80 mg/kg bwt until the last day of treatment. The animals were housed in standard mice cages at a temperature of 21–28°C, a 12 h light/dark cycle and provided ad libitum access to water and standard mice cubes obtained from Unga Feeds Ltd Kenya. Wood-chippings were provided as bedding material.

### *Trypanosoma brucei rhodesiense* infection and administration with MelB and GB

*Trypanosoma brucei rhodesiense*, KETRI 2537, initially established in Kenya, Lambwe Valley and Busoga, eastern Uganda known for HAT transmission was used in this study. Cryopreserved trypanosome stabilates in 50 μl capillary cryovial tubes were obtained from the trypanosome bank at the Biotechnology Research Institute (BIORI)-Muguga and left at room temperature to allow them to thaw. The viability of the parasites was confirmed microscopically before counting and infecting two donor mice. Thawed EDTA saline glucose (ESG) buffer was used to dilute the parasites after which viability was checked again, and donor mice were infected systematically to multiply the stock of the trypanosome stabilate after which each mouse was infected with 5.0 × 10^4^ trypanosomes. Melarsoprol with the strength of 180 mg/5ml administered intraperitoneally for 10 days at a dose of 2.2 mg/kg bwt after dilution with propylene glycol.

Oral administration of GB 80 mg/kg once daily was carried out using a gavage. This is the standard dose used in most studies and recommended by manufacturers [25]. Tanakan tablets (EGb 761) 40 mg (eCRATER USA) were utilized in this study. Each 40 mg tablet contains a dry extract from *Ginkgo biloba* leaves/ folium containing 22.0%-27.0% of flavonoids expressed as flavone glycosides, 5.4%-6.6% of terpene lactones, 2.8%-3.4% of ginkgolides A, B, C and 2.6%-3.2% of bilobalide, together with other excipients. The tablets were crushed using a clean mortar and pestle and dissolved at the ratio of 1 tablet in 5 mL of distilled water before giving the required dose.

### Determination of survival rate and parasitaemia

Survival experiments were carried out by monitoring mice daily for any clinical symptoms and signs, the time and the day at which each mouse succumbed were recorded in an Excel sheet and then analysed with Log-rank (Mantel-Cox) Test. Infected groups of mice were checked for the presence of parasitaemia. Mouse-tail blood was collected daily for the first week and thereafter every other two days. A drop of blood was placed on a clean slide, covered with a cover slip, and the parasites were counted using a light microscope. In order to assess the parasitaemia level, the matching technique developed by Herbert & Lumsden [26] was employed.

### Sample collection

After the treatment period, mice were euthanized with ketamine (50 mg/ml) and xylazine (100 mg/ml) (Merck KGaA., Darmstadt, Germany) in a ratio of 4:1 through intramuscular injection. Whole blood samples were collected intracardially, and placed in the EDTA tubes and used for haematological analyses. For biochemical test blood samples were collected in the Eppendorf tubes and the sample were centrifuged at 7826*g*, for 5 min at room temperature, and serum (supernatant) was collected. Serum was used for biochemical, nitrite, MDA and cytokine analyses. After the collection of blood intracardially, intracardial perfusion with 1X sterile PBS was done before harvesting of spleens, kidneys, livers, lungs, hearts and brains. These organs were used for GSH and MDA analysis. The organs were homogenized using homogenization buffer (0.25M Sucrose, 5 mM of Hepes-Tris and protease cocktail inhibitor) pH 7.4. The homogenates were frozen awaiting further analysis.

### Evans blue assay

To assess the stability of the blood brain barrier, Evans blue assay was performed. Mice were injected intravenously through the tail with 200μL of 1% Evans Blue dye prepared in sterile PBS. After one hour, mice were euthanized and brains extracted, photographed, weighed and then placed in falcon tubes containing 1ml of formamide and thereafter incubated for 48 hours at 37°C to extract Evan’s Blue dye from the tissues. The concentration of Evans blue was measured using a spectrophotometer at 620 nm.

### Determination of haematological values, organ function and electrolytes

Whole blood samples in EDTA tubes were analysed for complete blood count using an automated Benchman Coulter counter. The serum obtained was used to measure the levels of liver and kidney enzyme markers, albumin, lipid profile, electrolytes, creatinine, urea and uric acid levels. An automatic analyzer (Integra 400 plus analyzer, Roche Diagnostics) was used.

### Cytokine analysis

The serum levels of the pro-inflammatory cytokines i.e. TNF-α, IFN-γ and anti-inflammatory cytokines IL-10 were assessed via sandwich enzyme-linked immunosorbent assay (Thermo Fischer Scientific, California, USA) technique following the manufacturer’s guidelines. The concentration of cytokines was measured using an ELISA reader and the absorbance was taken at 450 nm.

### Reduced glutathione (GSH) assay

The levels of GSH from the brain, liver, spleen, heart, lungs and kidney were measured by employing the method of Griffith [27]. The organ homogenates were mixed with 5% w/v prepared solution of sulphosalicylic acid together with 0.25 mM EDTA. The brain, liver, kidney, heart, lungs and spleen homogenate samples were mixed with Ellman’s reagent. The absorbance of the reaction was determined spectrophotometrically using a microplate reader (absorbance set at 405 nm).

### Quantification of nitric oxide using Griess assay

Nitric oxide (NO) was quantified as nitrite. Nitrates were reduced to nitrites by enzymatic conversion by nitrate reductase (SIGMA). The level of nitrites was determined by the Griess method. NO production was then quantified by measuring colour change at 550 nm using a spectrometer (Spectra Max 340pc384, Molecular Devices, Sunnyvale, USA).

### Assessment of Malondialdehyde (MDA)

To determine lipid peroxidation levels in mice sera and organs, MDA levels were measured by assays of thiobarbituric acid reactive species (TBARS) [Draper and Hadley [28]. Serum, kidney, liver, spleen and brain samples were mixed with an equal volume of thiobarbituric acid 0.67% and heated at 92–96°C for 30 min. Thiobarbituric acid reactive species production was quantified at 535nm using a spectrometer. Results were expressed as malondialdehyde per milligram of protein.

### Statistical analysis

One-way ANOVA was done to test the difference among different groups of data with Tukey’s posthoc test for internal comparison. The mean survival time was analysed by the use of the Log-rank (Mantel-Cox) Test. The statistical difference in the mean levels of parasitaemia was determined by t-test. Results were reported as ± SEM with levels of significance set at P<0.05.

## Results

### Co-administration of MelB and GB resulted in improved survival rate in the course of T.b.r infection

**Fig 1:**
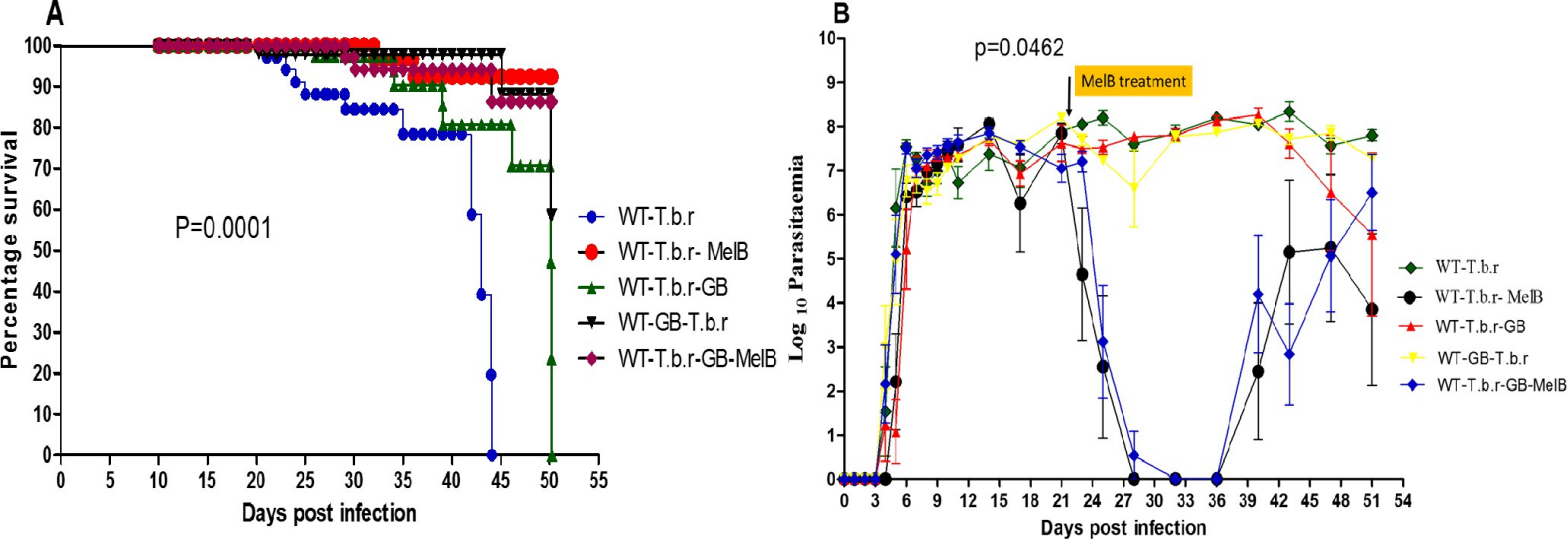
Survival rate, parasitaemia and effect of administration of MelB and GB on the blood brain barrier integrity during T.b.r infection in mice. The figures show the percentage survival rate (**A**) and parasitaemia levels (**B**). Comparison between groups was done using One-way ANOVA with Tukey’s test for multiple comparisons.

Wild type T.b.r + GB group had a statistically significant (P<0.001) improvement in survival rate, with a median of 50 days post-infection, compared to the wild type T.b.r infected group which had a median survival of 43 days post-infection. (P<0.0001) (Fig 1A). All the mice in the T.b.r infected but untreated group had succumbed to the infection by the 44^th^ day post-infection. On the other hand, mice that were infected and supplemented with GB survived for a longer duration, up to 50 days post infection. Notably, all the surviving mice were sacrificed in order to minimize suffering due to high parasitaemia and anaemia. Additionally, results from the study revealed comparable parasitaemia levels for all treatment groups except for MelB treated (p>0.05). The first peak of parasitaemia was observed in the initial phase post- infection between day 4 and 8 for all experimental groups of mice (Fig 1B). This was followed by cycles of varying parasitaemia across all the groups until the last day of the experiment. However, mice in the infected group had the highest parasitaemia by the time of the termination of the experiment. After MelB administration, there was a sharp decline in parasitaemia in the groups treated with MelB alone or with GB, although this was followed by a steady increase in parasitaemia, most likely due to relapse.

### Exposure to MelB and GB prevented damage to the blood brain barrier in infected mice

**Fig 2:**
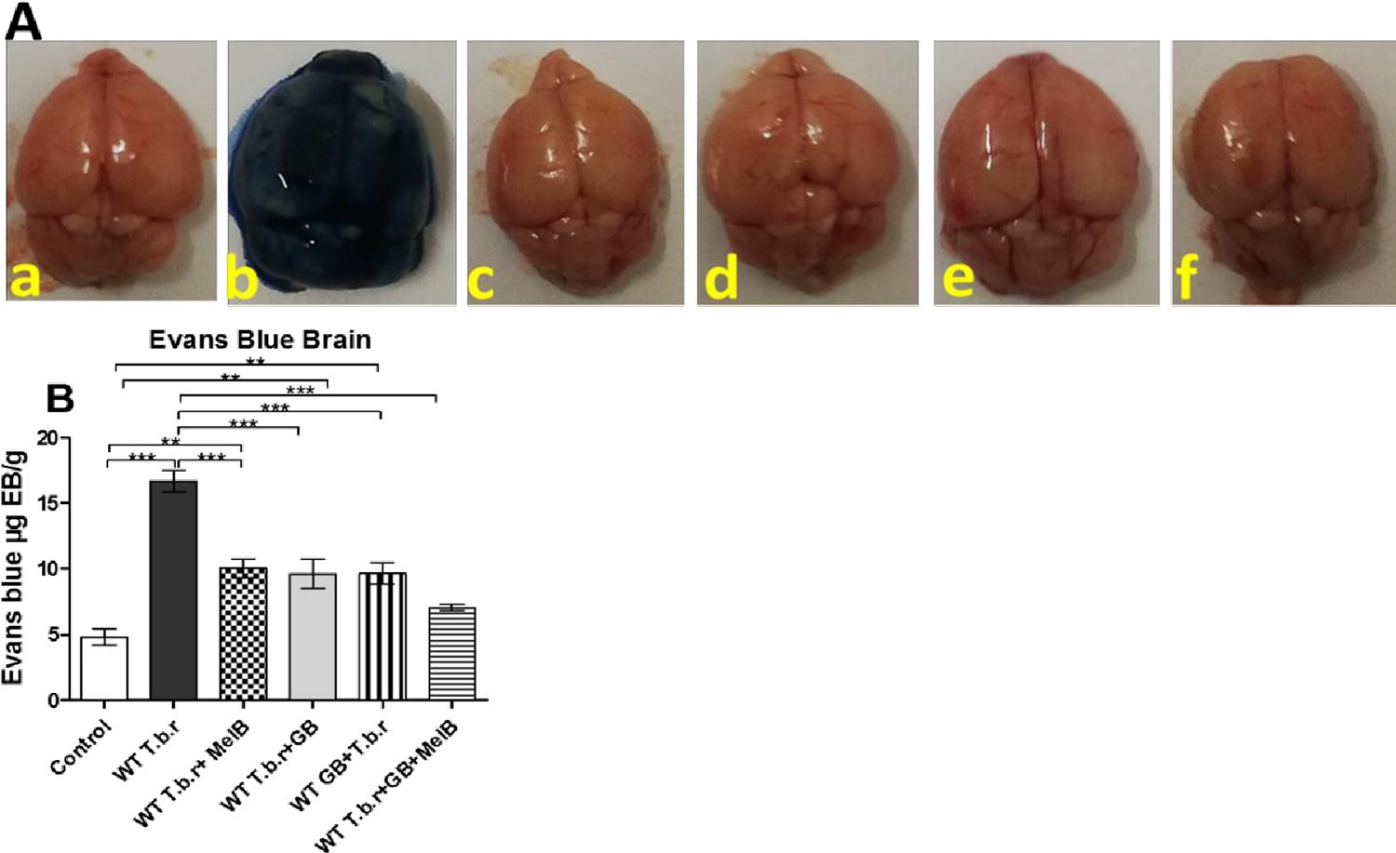
Effect of administration of MelB and GB on the blood brain barrier integrity during T.b.r infection in mice. The figures show the uptake of the Evans Blue dye (**A**) by the brain tissue and quantification of Evans blue dye extravasated (**B**). Comparison between groups was done using One-way ANOVA with Tukey’s test for multiple comparisons (Asterisks indicates levels of significant differences: **p<0.01***p<0.001).

Evans blue assay was performed in order to establish the integrity of the BBB during T.b.r infection. Evans blue dye binds to serum albumin. The results revealed extravasation of the Evans Blue dye in brains of mice infected with T.b.r as opposed to those in the normal control group (Fig 2A); indicating damage and breach of the blood-brain barrier. Notably, the amount of Evans Blue dye that penetrated the brains of mice administered with GB and MelB were remarkably lower (p<0.001) in contrast with T.b.r infected mice (Fig 2B). Notably, even in the absence of MelB, GB preserved the integrity of the blood-brain barrier in infected mice.

### Administration of MelB and GB attenuated T.b.r-induced microcytic hypochromic anaemia

**Fig 3:**
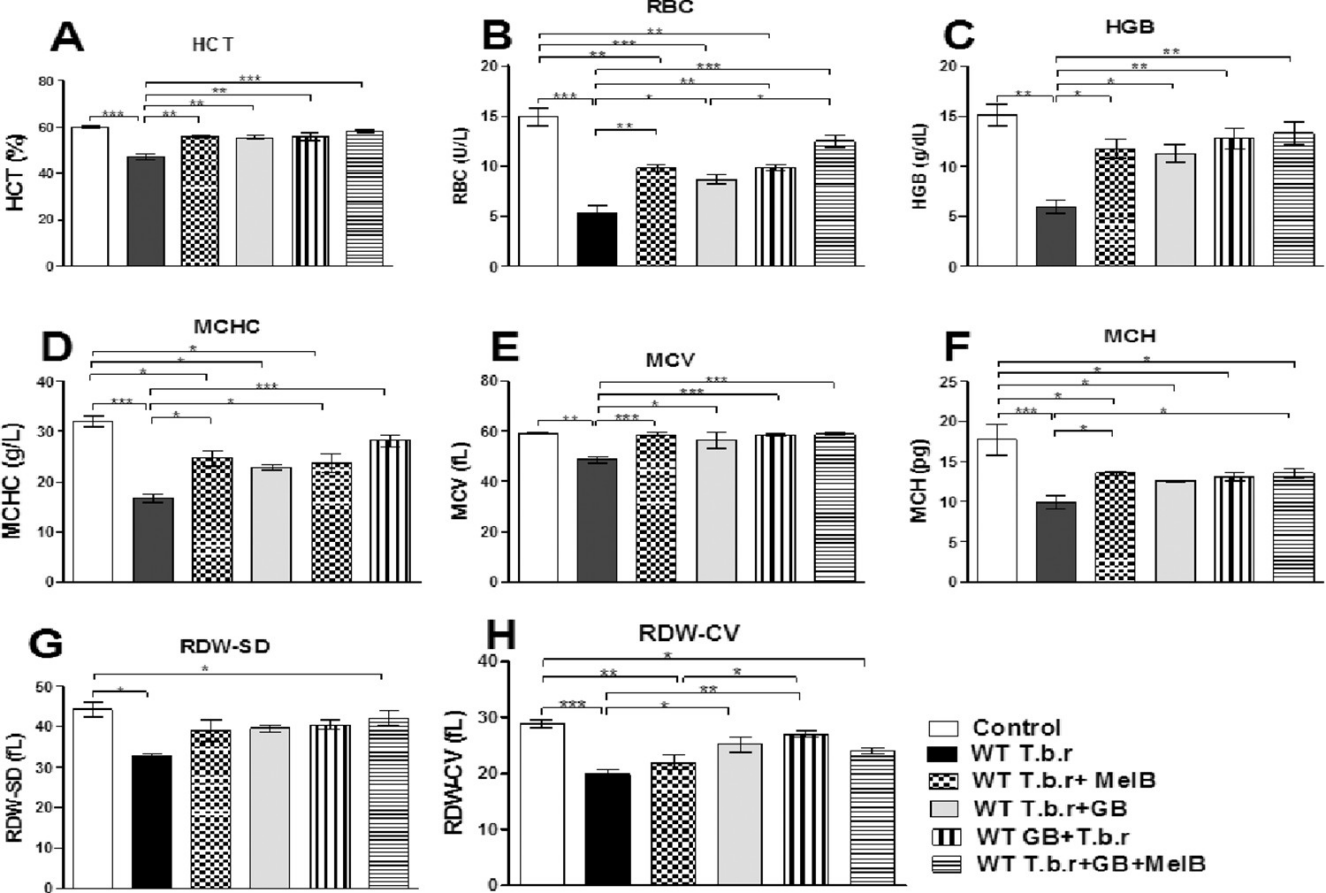
Impact of treatment with MelB and GB on variations brought about by T.b.r in HCT, HGB, RBC and red cell indices. The figures shows the levels of blood HCT (**A**), RBC (**B**), HGB (**C**), MCHC (**D**), MCV (**E**), MCH (**F**), RDW-SD (**G**) and RDW-CV (**H**). Comparison between groups was done by One way ANOVA followed by Tukey’s posthoc test. (Indicated level of significance: *p<0.05 **p<0.01 ***p<0.001).

Mice infected with T.b.r had significantly decreased haematocrit (HCT) (p<0.001, Fig 3A), and RBC (p<0.01, Fig 3B) levels accompanied by low haemoglobin levels (p<0.05, Fig 3C) when compared to those in the control group; suggesting that T.b.r infection causes anaemia. Notably, administration of GB and MelB separately nullified the T.b.r-induced suppression of haematocrit, RBC and haemoglobin when comparisons are made with the infected (untreated) and normal (uninfected) control. In the presence of GB and MelB, RBC levels and those of HCT and HGB were comparable to the control, indicating a robust protective effect from T.b.r-induced anaemia. In order to find out the specific type of anaemia, the levels of various RBC indices were assessed. Mice infected with T.b.r had low levels of Mean corpuscular volume MCV (p<0.01, Fig 3D), Mean corpuscular haemoglobin MCH (p<0.01, Fig 3E), Mean corpuscular haemoglobin concentration MCHC (p<0.01, Fig 2F), RDW-SD (p<0.05, Fig 3G) and RDW-CV (p<0.01, Fig 3H). These features are characteristic of microcytic hypochromic anaemia. When MelB and GB were administered separately, the suppression of all the blood indices by T.b.r was blocked. This, therefore, implies that GB, MelB and co-exposure to GB with MelB averted T.b.r- induced microcytic hypochromic anaemia. Notably, there was no significant difference between T.b.r+MelB versus T.b.r+MelB+GB in regard to suppression of blood indices across the board.

### High white blood cell count was recorded when GB was administered alone or with MelB during T.b.r infection

**Fig 4:**
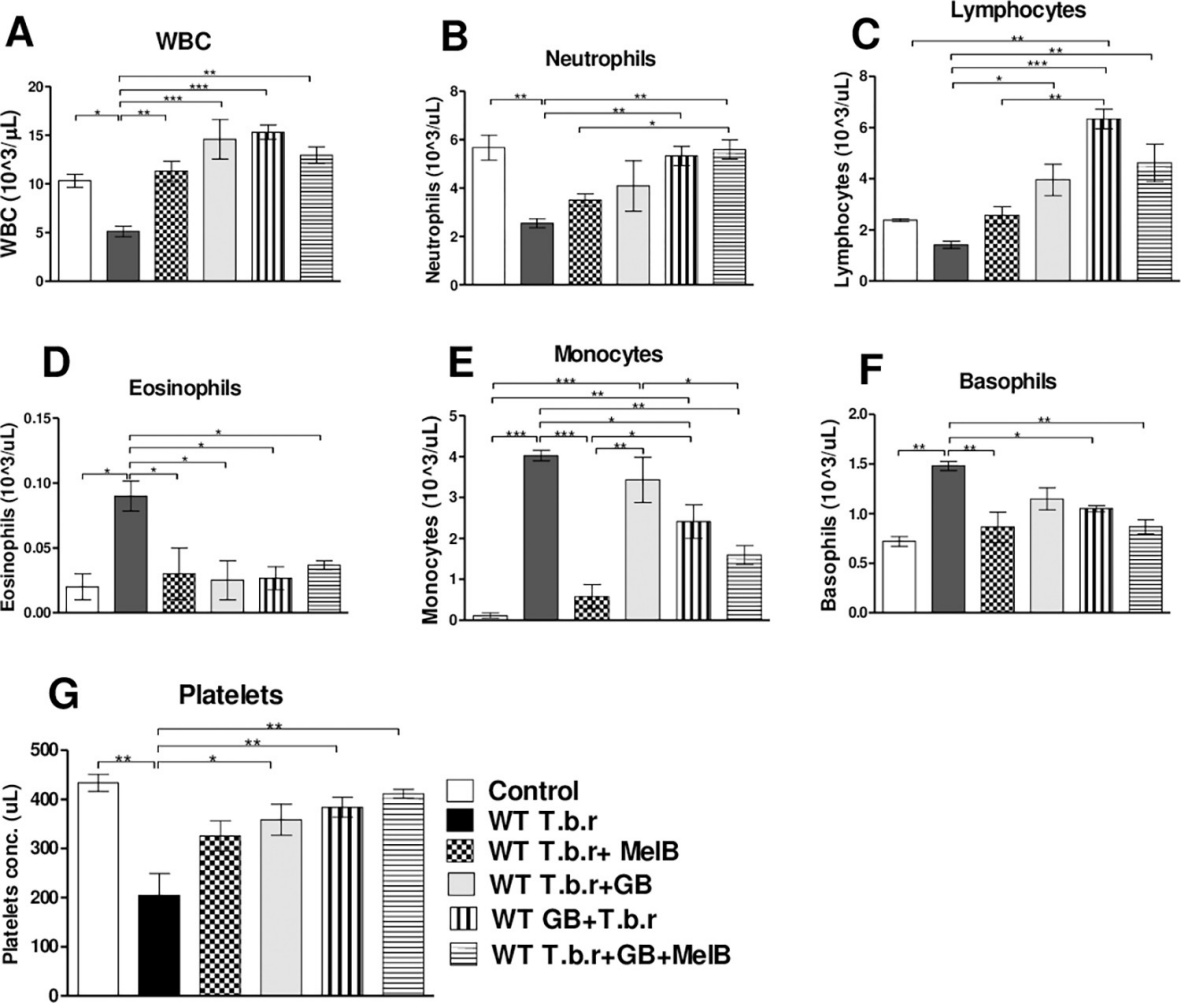
Effect of administration of MelB and GB on WBC and WBC subtypes in T.b.r infected mice. The figure shows the levels of blood WBC (**A**), neutrophils (**B**), lymphocytes (**C**), eosinophils (**D**), monocytes (**E**), basophils (**F**) and platelets (G). Comparison between groups was done using One-way ANOVA followed by Tukey’s posthoc test. (Indicated level of significance: *p<0.05 **p<0.01 ***p<0.001).

There was a decrease in total WBC in mice infected with T.b.r compared to those in the control group (p<0.05). However, mice administered with GB had a significant increase (p<0.01) in WBC relative to those infected with T.b.r. Similarly, mice co-administered with GB and MelB recorded a significant increase (p<0.05) in WBC as compared to the infected group (Fig 4A). The levels of total WBC were comparable between MelB treated alone and the group of mice co-exposed to GB and MelB. The study demonstrated significant T.b.r-driven decline in neutrophils (p<0.01, Fig 4B). T.b.r infection did not significantly change the level of lymphocytes (Fig 4C). However, GB significantly drove elevation of lymphocytes in infected mice. On the other hand, the infected mice had elevated levels of eosinophils (Fig 4D), monocytes (p<0.001, Fig 4E) and basophils (p<0.05, Fig 4F). Notably, co-administration with GB and MelB resulted in a significant increase in neutrophils when compared to T.b.r+MelB (p<0.05) and a decrease in monocytes and basophils (p<0.05) when compared to the infected group. Inoculation of mice with T.b.r led to a notable decrease (p<0.05) in platelets when compared to the control group (Fig 4G). GB protected from T.b.r-driven depletion of platelets. MelB, when administered alone, did not significantly prevent T.b.r-driven suppression of platelets. In the presence of GB (T.b.r+MelB+GB), platelet levels were stabilized (p<0.05), and comparable to the control. In this study, it is clear that GB as an adjuvant therapy to MelB, has the capacity to prevent thrombocytopenia in late-stage HAT.

### Effect of MelB and GB on lipid metabolism in T.b.r infected mice

**Fig 5:**
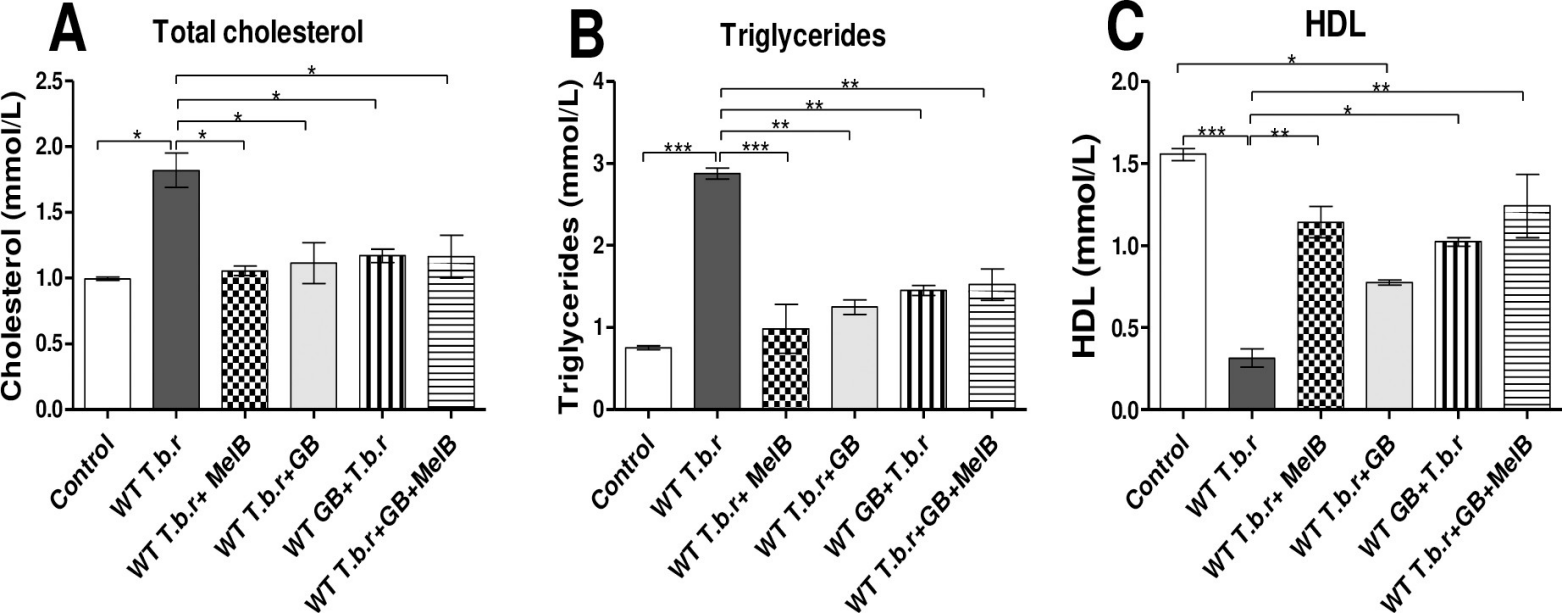
Effect of MelB and GB administration on lipid metabolism in HAT. The figures show serum levels of total cholesterol (**A**), triglycerides (**B**) and HDL (**C**). Comparison between groups was done using One-way ANOVA with Tukey’s posthoc test for internal comparisons. (Indicated level of significance: *p<0.05 **p<0.01 ***p<0.001).

Infection with T.b.r significantly increased total cholesterol (p<0.05, Fig 5A) and triglycerides (p<0.01, Fig 5B) levels relative to the control group. However, the infected mice had significantly low HDL (p<0.01, Fig 5C) compared to the control. Notably, administration of GB and MelB blocked the T.b.r-induced dyslipidemia. However, there was no significant difference between T.b.r+MelB and T.b.r+MelB+GB.

### GB protected the brain, spleen, heart and lungs from T.b.r and MelB-induced changes on GSH levels

**Fig 6:**
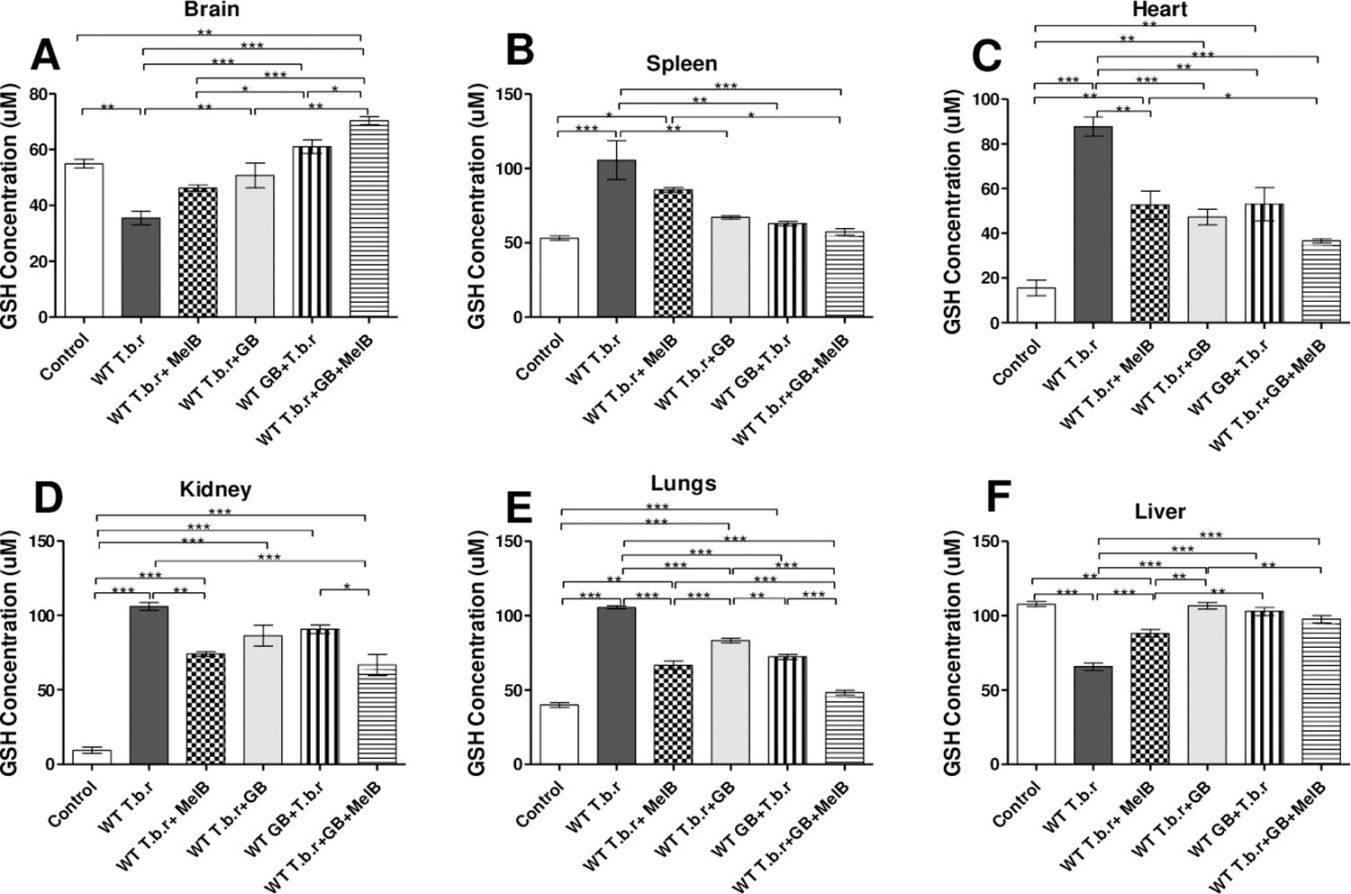
The effect of T.b.r infection and administration of MelB and GB on cellular GSH levels in different organs. The figures show the cellular GSH levels from brain (**A**), spleen (**B**), heart (**C**), kidney (**D**), lungs (**E**) and liver (**F**). Comparison between groups was done using One-way ANOVA with Tukey’s posthoc test for internal comparisons. (Indicated level of significance: *p<0.05 **p<0.01 ***p<0.001****p<0.0001).

T.b.r infection caused a significant decrease in brain GSH (p<0.05, Fig 6A) relative to the control group and those treated with either MelB or GB. MelB administration, when compared to the control and the infected (untreated), significantly elevated GSH levels in the spleen and heart (Fig 6B & 6C). Co-administration of MelB and GB (T.b.r+MelB+GB) was significantly effective in stabilizing GSH levels when compared to MelB (T.b.r+MelB) for the brain, spleen, heart and lungs. Overall, administration of GB had a positive effect in stabilizing GSH levels for the brain, spleen and liver. Notably, GB significantly blocked T.b.r and MelB-driven depletion of GSH in the brain as well as its elevation in the spleen. It was also noted that infection with T.b.r led to a significant GSH increase in the spleen (p<0.01, Fig 6B), heart (p<0.001, Fig 6C), kidney (p<0.001, Fig 6D) and lungs (p<0.001, Fig 6E) as compared to the control group. Co-administration (T.b.r.+MelB+GB) was more effective in blocking T.b.r-driven changes to GSH levels for the brain, spleen and heart when compared to T.b.r+MelB. Moreover, T.b.r and MelB-driven depletion of hepatic GSH levels was blocked by GB (p<0.001, Fig 6A). GB provided superior protection to GSH levels in the presence of T.b.r across all organs investigated (Fig 6A–6F) when comparisons are made with the normal control and infected (untreated) control.

### The effect of T.b.r, MelB and GB on MDA levels

**Fig 7:**
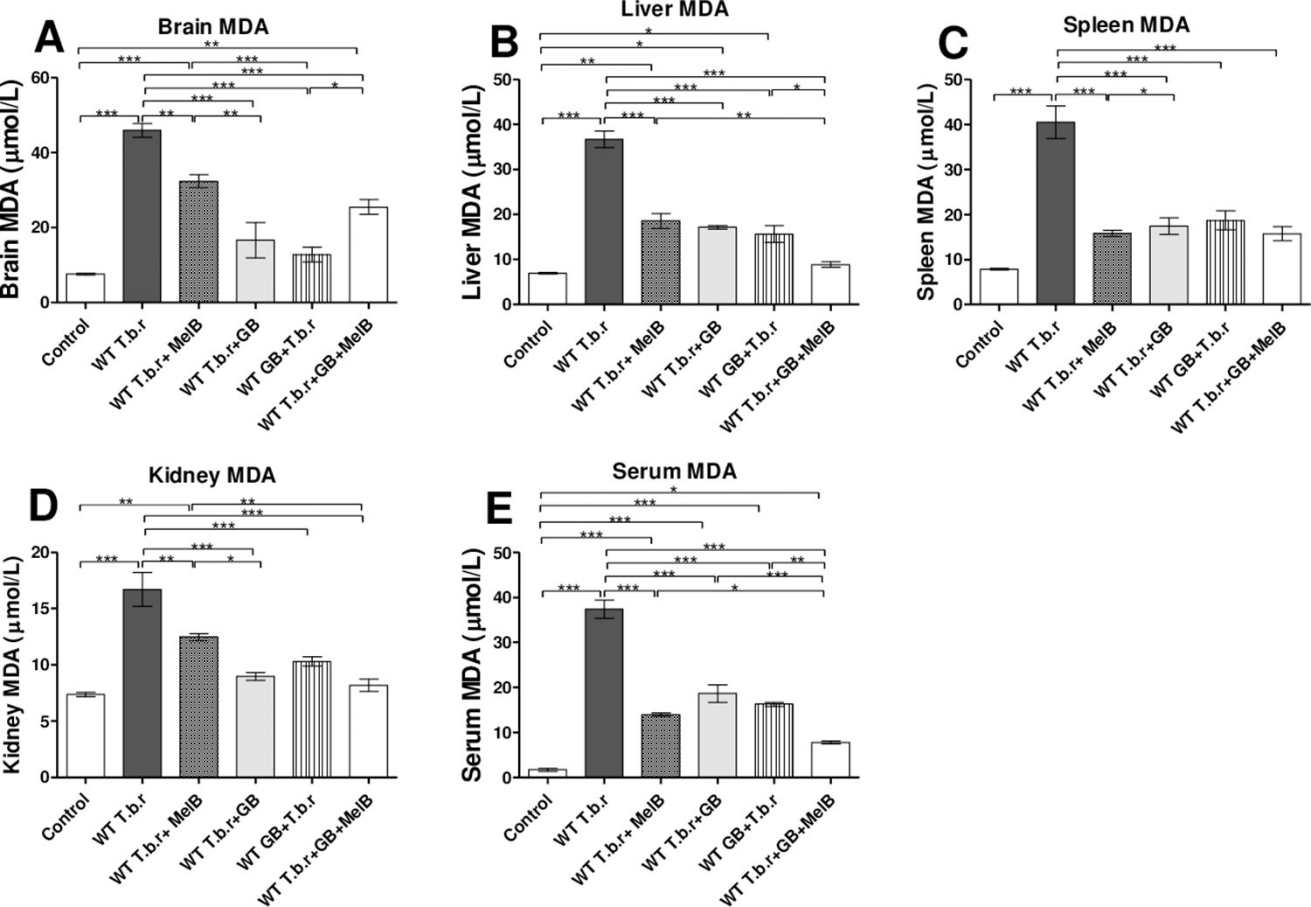
The effect of T.b.r infection, MelB and GB administration on MDA levels during HAT. The figures show the MDA levels from brain (**A**), liver (**B**), spleen (**C**), kidney (**D**) and serum (**E**). Comparison between groups was done using One-way ANOVA with Tukey’s posthoc test for internal comparisons. (Indicated level of significance: *p<0.05 **p<0.01 ***p<0.001).

Mice infected with T.b.r had remarkably high levels of MDA in the brain (p<0.001, Fig 7A), liver (p<0.001, Fig 7B), spleen (p<0.001, Fig 7C) and kidney (p<0.05, Fig 7D), relative to those in the normal control group. Serum MDA were significantly elevated (p<0.001, Fig 7E) in T.b.r infected mice when compared to the control. It is also notable that MelB administration resulted in significant elevation of MDA in the brain, liver and kidney (Fig 7A, 7B and 7D). Elevation of MDA is a characteristic indicator of active lipid peroxidation. Notably, co-administration of GB and MelB (T.b.r+MelB+GB) and GB (alone) significantly attenuated T.b.r and MelB driven elevation of MDA for the liver and serum (Fig 7B and 7E). MDA levels for the brain and kidney demonstrate superior protection from GB, when compared to MelB.

### GB stabilized T.b.r and MelB-driven rise in nitrite

**Fig 8:**
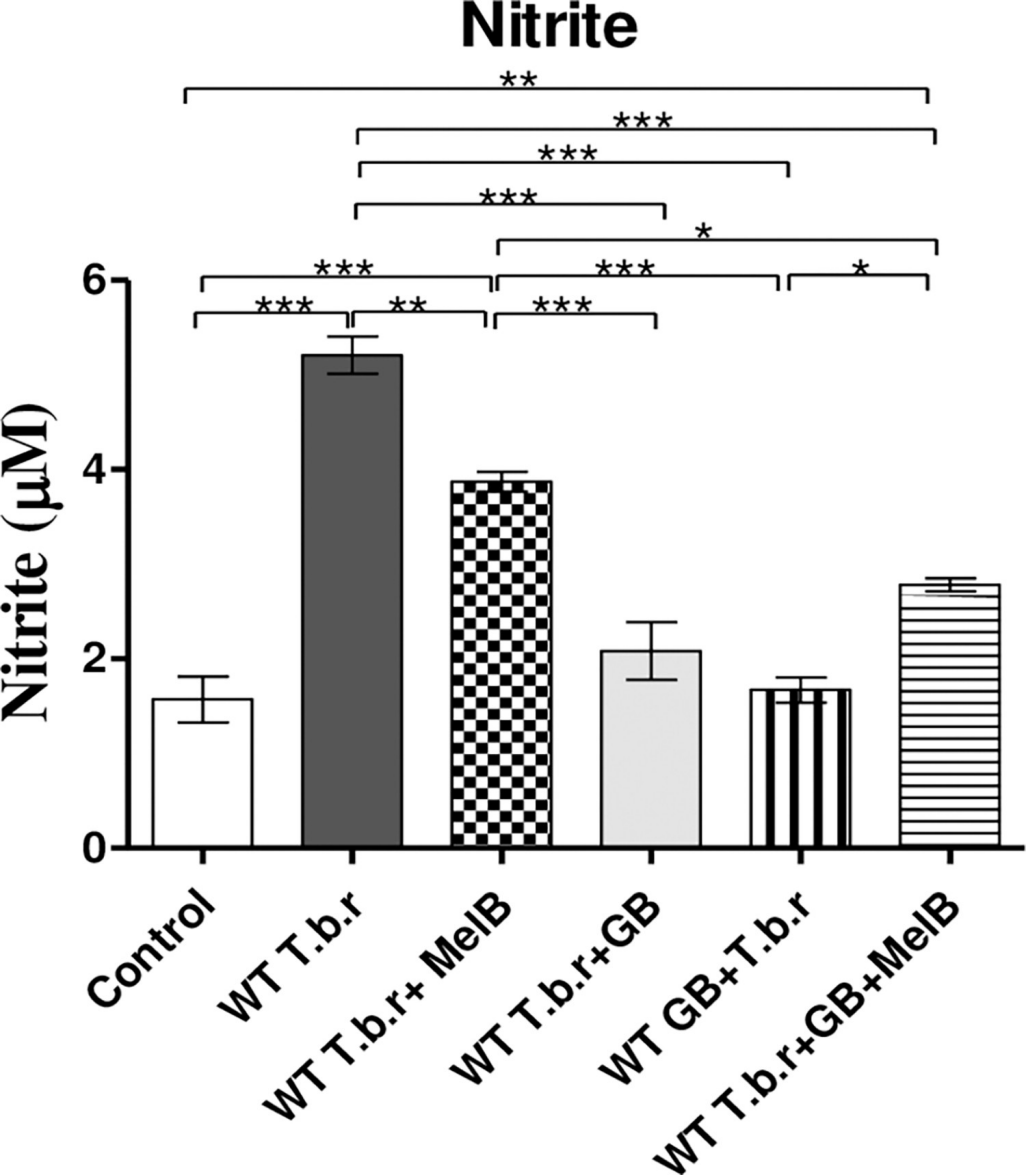
The effect of T.b.r infection, MelB and GB administration on nitrite levels. Comparison between groups was done using One-Way ANOVA with Tukey’s posthoc as posteriori test. (Indicated level of significance: *p<0.05 **p<0.01 ***p<0.001).

There was a notable elevation in nitrite level (p<0.001, Fig 8) in mice infected with T.b.r as compared to those in the control group. When compared to the control, MelB significantly raised nitrite levels On the other hand, GB significantly attenuated T.b.r and MelB-driven elevation of nitrite when administered separately or when co-administered (T.b.r+MelB+GB).

### Co-administration of MelB and GB stabilized TNF-α and IFN-γ levels in T.b.r infected mice

**Fig 9:**
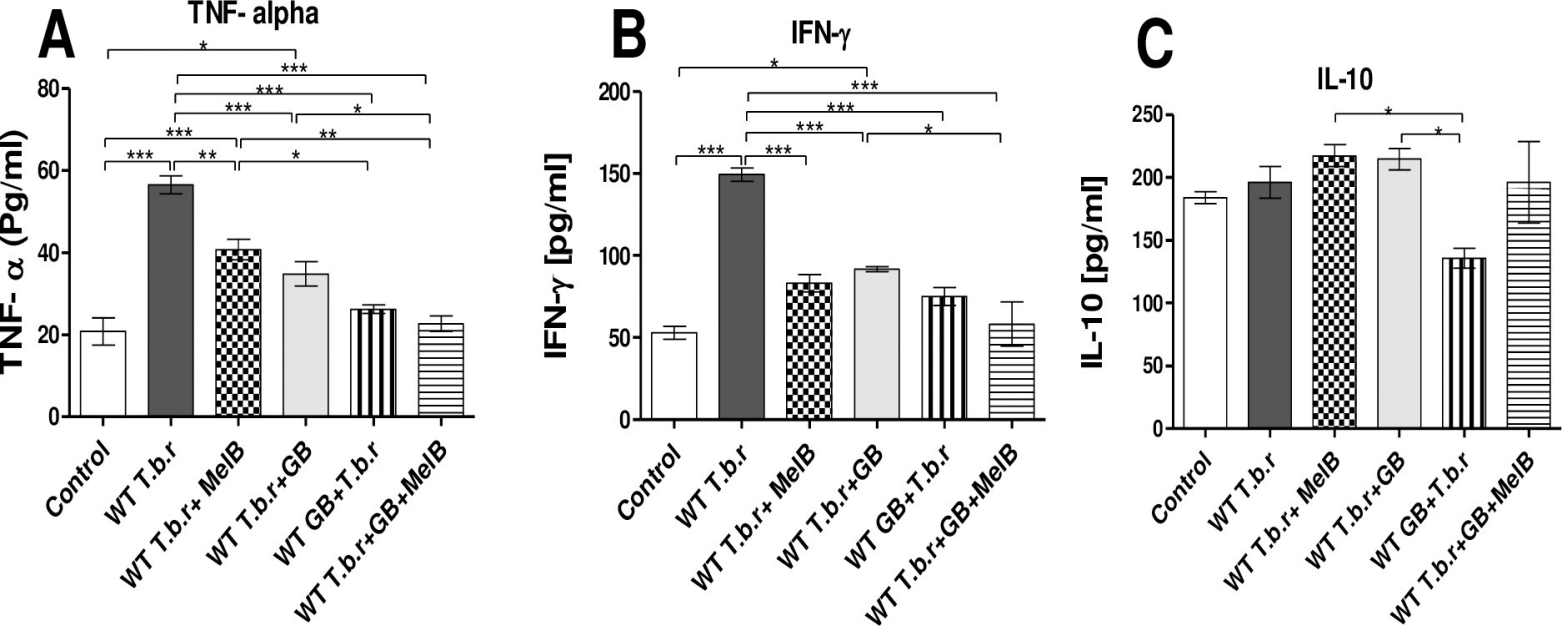
Levels of pro-inflammatory and anti-inflammatory cytokines in mice infected with T.b.r and treated with MelB and GB. The figures show the serum levels of pro-inflammatory cytokines TNF-α (**A**), IFN-γ (**B**) and anti-inflammatory cytokine IL-10 (**C**). Comparison between groups was done using One-way ANOVA with Tukey’s posthoc used for internal comparisons. (Indicated level of significance: *p<0.05 **p<0.01 ***p<0.001).

The infected mice showed a remarkable increase in serum TNF-α (p<0.001, Fig 9A) as well as IFN-γ (p<0.001, Fig 9B) relative to those in the control group; an indication of inflammation. Notably, the levels of TNF-α and IFN-γ decreased in infected mice treated with MelB and administered with GB before infection and those where Mel B and GB were co-administered. Treatment with either MelB or GB separately, attenuated T.b.r-induced elevation of pro-inflammatory cytokines IFN-γ and TNF-α. Additionally, the elevation of the pro-inflammatory cytokines TNF-α and IFN-γ was blocked (p<0.001) by co-administration of GB and MelB; when compared with mice infected with T.b.r or treated with only MelB. Moreover, a group of infected mice treated with GB had comparable levels of IL-10 relative to the normal control. Administration of GB before infection with T.b.r significantly reduced the anti-inflammatory cytokine IL-10 levels (p<0.05, Fig 9C).

### The effect of T.b.r infection and administration of MelB and GB on liver function

**Fig 10:**
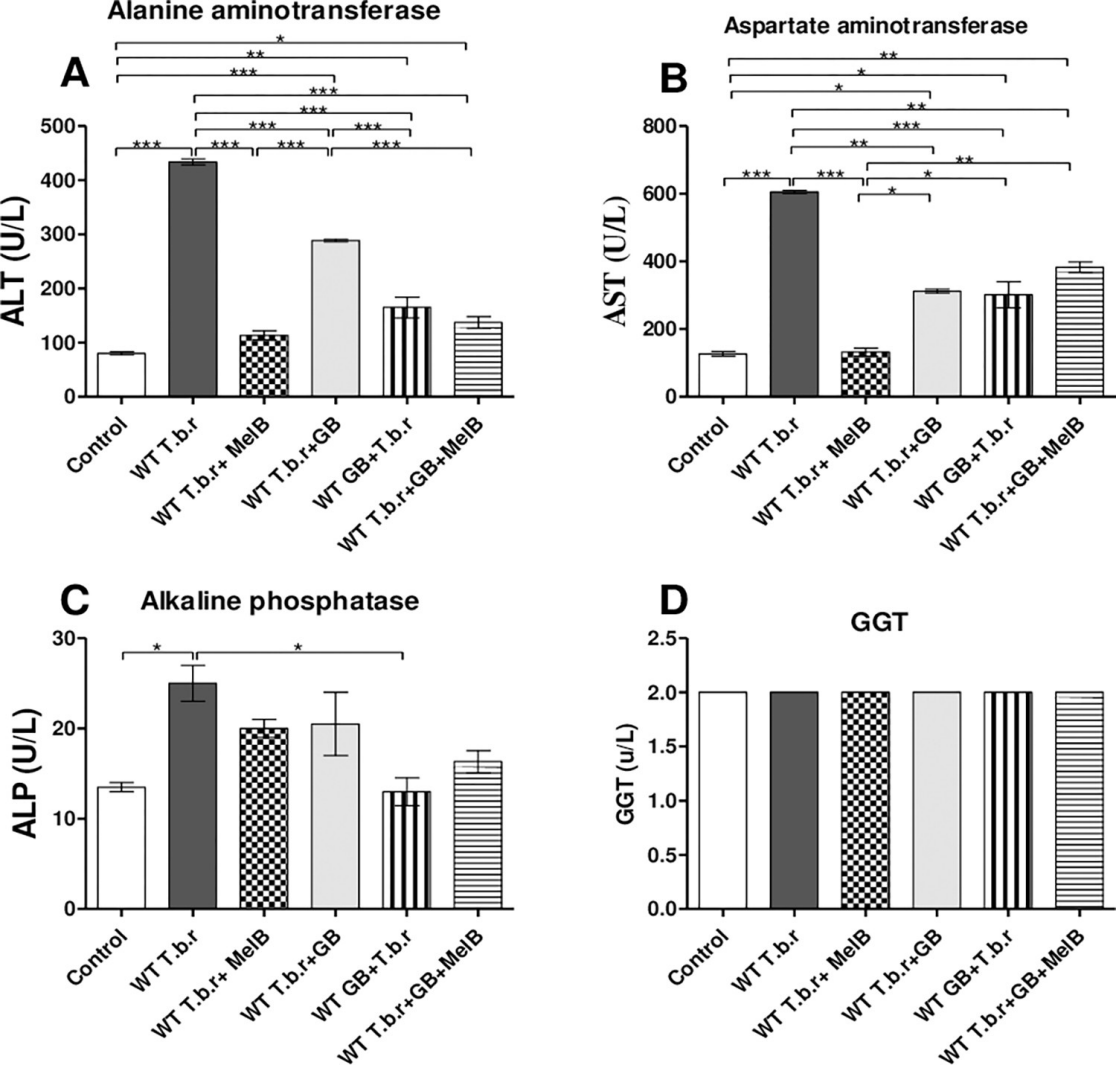
The effect of T.b.r infection and administration of MelB and GB on liver function biomarkers (ALT, AST, ALP and GGT) in the course of T.b.r infection. The figures show the serum levels of ALT (**A**), AST (B), ALP (**C**) and GGT (**D**). Comparison between groups was done using One-way ANOVA with Tukey’s test as posteriori test. (Indicated level of significance: *p<0.05 **p<0.01 ***p<0.001).

Mice infected with T.b.r had significantly high levels of ALT (p<0.001, Fig 10A), AST (p<0.001, Fig 10B) and ALP (p<0.05, Fig 10C) relative to the normal control group. The heightened levels of these biomarkers signified active liver damage. A comparative analysis of MelB or GB treatment to T.b.r infected mice and the normal control, revealed a significant normalization of the serum levels of ALT (p<0.001) and AST on exposure to MelB or GB. Additionally, there was a significant abrogation of T.b.r-driven elevation of AST (p<0.001) in infected mice co-administered with GB and MelB (T.b.r+MelB+GB) in comparison to MelB (T.b.r+MelB) treated mice (Fig 10B). Only GB was able to significantly attenuate T.b.r-driven elevation of AP. However, there was no statistical difference in GGT levels across all experimental groups (Fig 10D).

### The effect of T.b.r infection and administration of MelB and GB on urea, uric acid and albumin

**Fig 11:**
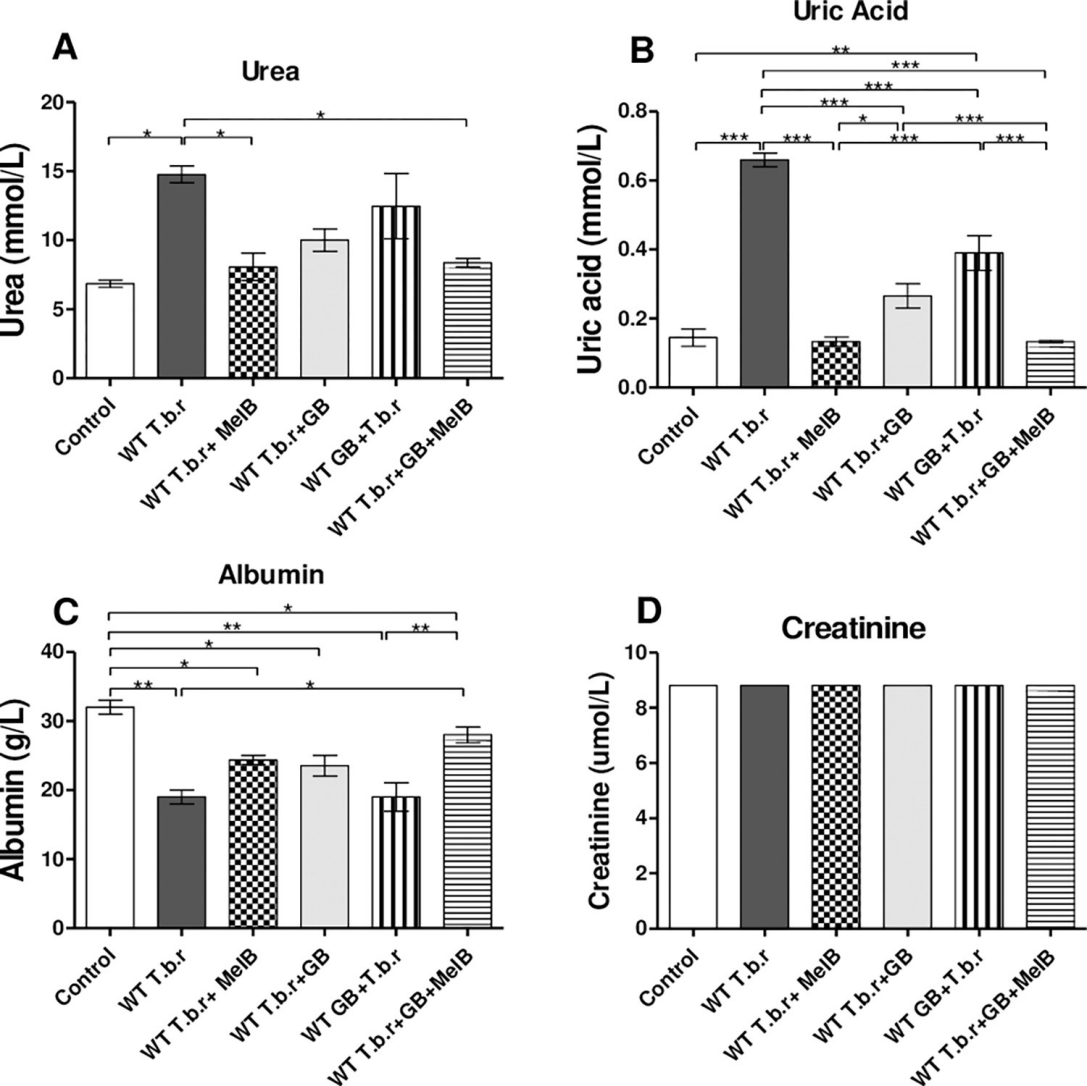
Effect of T.b.r infection and administration of GB and MelB on the serum levels of urea, uric acid, albumin and creatinine during HAT. The figures show the serum levels of urea (**A**), uric acid (**B**), albumin (**C**) and creatinine (**D**). Comparison between groups was done using One-way ANOVA with Tukey’s posthoc test for multiple comparisons. (Indicated level of significance: *p<0.05 **p<0.01 ***p<0.001).

Elevated levels of urea (p<0.05, Fig 11A) and uric acid (p<0.001, Fig 11B) were observed in mice infected with T.b.r when compared to those in the normal control group. MelB was the most effective in countering T.b.r-driven rise in urea and uric acid when compared to GB. Significantly low albumin levels (p<0.05, Fig 11C) were observed in infected mice, when compared to those in the normal control group; a phenomenon that was blocked when GB was given together with MelB. However, no statistical difference was observed in creatinine levels across all experimental groups (Fig 10D).

### Co-administration of Ginkgo biloba and melarsoprol prevented metabolic acidosis in mice infected with T.b.r

**Fig 12:**
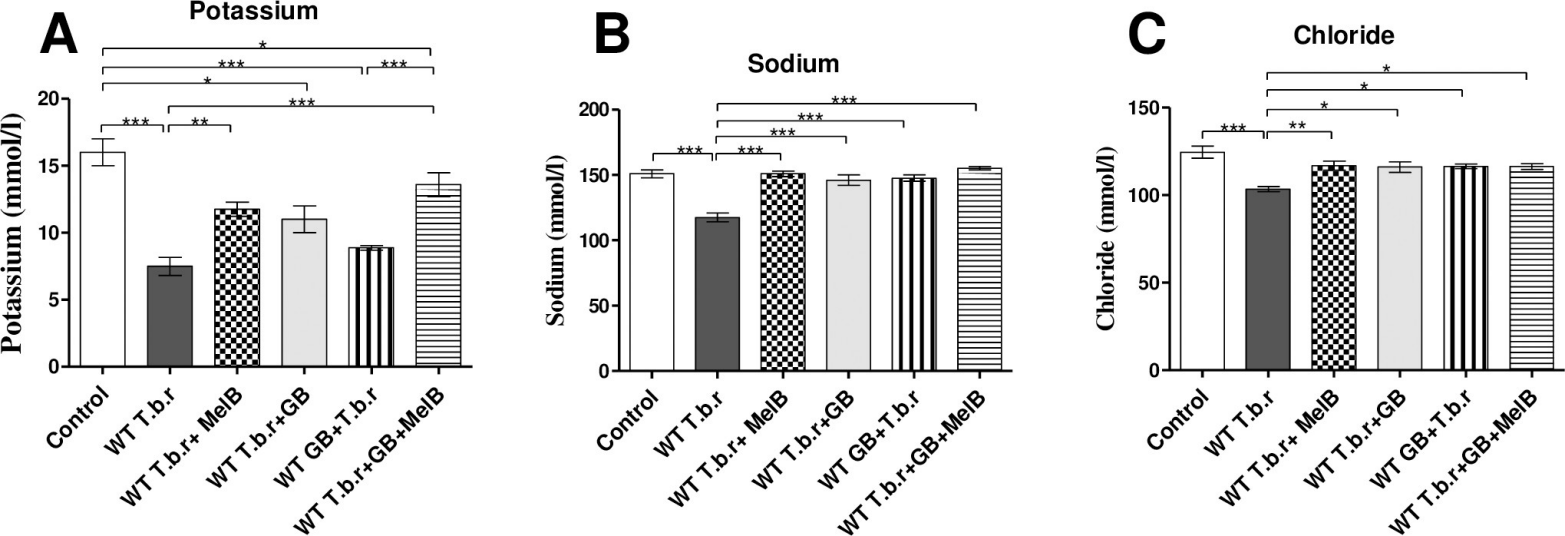
Impact of co-administration of MelB and GB on the serum levels of electrolytes (potassium, sodium and chloride) in T.b.r infected mice. The figures show the serum levels of potassium (**A**), sodium (**B**) and chloride (**C**). Comparison between groups was done using One-way ANOVA with Tukey’s posthoc test as posteriori test. (Indicated level of significance: *p<0.05 **p<0.01 ***p<0.001).

T.b.r infection led to the decrease of potassium (p<0.01, Fig 12A), sodium (p<0.001, Fig 12B), and chloride (p<0.01, Fig 12C) relative to the normal control group. This signified possible electrolyte disturbance i.e. metabolic acidosis. A significant increase of potassium (p<0.001), sodium (p<0.0001) and chloride (p<0.01), was noted following acute exposure to MelB relative to T.b.r infected mice. Notably, GB alone or in combination with MelB prevented the electrolyte imbalance caused by T.b.r infection.

## Discussion

The current study demonstrates that oral administration with GB ameliorates negative biochemical and physiological events caused by T.b.r during severe HAT. Notably, GB prevents damage to the blood-brain barrier due to T.b.r and reduces oxidative stress and inflammation caused by both the trypanosomes and melarsoprol. Another notable finding was that GB led to an improved survival rate of mice infected with T.b.r. The capacity of GB to boost the survival rate in T.b.r infected mice validates other studies with similar findings. For example, in one such study, GB improved the survival rate in a mouse model after induction of sepsis with an inflammatory agent, lipopolysaccharide [29]. The increased survival rate in mice administered with GB may be attributed to its anti-inflammatory and antioxidant properties; that probably delay the onset of pathophysiology noted during late-stage HAT. Parasitaemic waves were evident in this study because the parasite is able to dodge the host immune system [30–32]. The ability to evade the immune system by the parasite is due to the antigenic variation of the parasite’s variant surface glycoprotein (VSG) [33]. At the initial phase of parasitaemia, most of the parasites are homotype of similar antigenic categories. This homotype is identified by the host immune system, and consequently, antibodies are produced [34]. This is then followed by a declining phase due to the elimination of the parasites of the major variable antigenic type (VAT). Nonetheless, the minor VATs (heterotype) continue to multiply and eventually become dominant. This phenomenon leads to parasitaemia waves and enduring chronic infection [34].

*Trypanosoma brucei* alters brain function by penetrating the blood-brain barrier and accumulating in the brain [35]. Breach of the blood brain barrier is a major event in the pathogenesis of HAT due to T.b.r; and is associated with a myriad of negative, devastating effects on the CNS. MelB and GB were equally effective in preventing T.b.r-induced damage to the blood brain barrier. For GB, this observation may be attributed to the flavonoids in GB, that act as free radical scavengers, while terpene lactones safeguard mitochondrial membranes of neuronal cells from harm caused by free radicals, known to cause oxidative stress and neuronal damage [36]. Gohil and Packer [37] assert that EGb 761 is capable of scavenging hydroxyl radicals since it possesses superoxide dismutase-like activity. Recently, it was established that a standardized extract of *Ginkgo biloba*, EGb 761, has neuroprotective effects in numerous central nervous systems and neurodegenerative diseases such as Parkinson’s and Alzheimer’s [38].

The meningoencephalitis stage of HAT is characterised by the penetration of the blood-brain barrier by trypanosomes and their multiplication in the cerebral spinal fluid and eventually aggregating in the brain parenchyma [39–40]. This finding was corroborated by our Evans Blue Assay data, with more dye accumulating in the brain of mice infected with T.b.r. The amount of Evans blue dye that penetrated the brains of mice co-administered with GB and MelB was lower, even though this change was not statistically significant. It will be of great interest to determine if dose response studies utilizing MelB and GB combinations would register greater beneficial effects when co-administered in regard to protection of the blood brain barrier. Further studies in this realm are desirable. The noted protective effects of GB may be attributable to Ginkgolide B, a constituent of EGb 761, which has been shown to reduce the permeability of the blood-brain barrier, in cerebral edema [41–42]. Nevertheless, the molecular mechanisms underlying the capability of GB to protect the breach of the blood-brain barrier requires further scrutiny.

Various physiological and biochemical processes are ravaged by HAT infection. These include blood components, resulting in anaemia [43]. Erythrocyte cell damage occurs in the course of HAT because of overwhelming oxidative stress [44]. This study confirmed the presence of trypanosomiasis-mediated anaemia, marked by low counts of RBC, HCT and haemoglobin. The current study corroborated a previous study by Neves et al. [45] which demonstrated the presence of microcytic hypochromic anaemia during T.b.r infection. In this study, it was demonstrated that MelB and GB when administered separately, improved T.b.r-induced anaemia. Co-administration of MelB and GB though beneficial, did not result in enhanced synergistic or additive pharmacological effect superior to those noted when administered separately in regard to blood indices. Future dose response studies may be able to establish if specific higher or lower doses may result in a significant effect when MelB and GB are co-administered. This constitutes an interesting area for scrutiny for future studies. It has been shown previously that *Ginkgo biloba* leaf extract has a protective role on red blood cells against Aβ- and hypotonic pressure-induced haemolysis, peroxide-induced lipoperoxidation, as well as glutathione consumption and methaemoglobin formation [46]. It is therefore likely that these aforementioned properties played a role and possibly protected the erythrocytes and prevented anaemia.

Infection with trypanosomes elicits very strong immune responses. The interaction between the parasite and the host immune system is driven by the variant surface glycoprotein (VSG), which is found on the membrane of the trypanosomes [33]. Infection with T.b.r resulted in significant downregulation of total WBCs, neutrophils, lymphocytes and platelets. On the other hand, eosionophils, monocytes and basophils were significantly elevated. Administration of MelB and GB restored T.b.r driven depletion of WBCs, neutrophils and lymphocystes, while decreasing the T.b.r-induced elevation of eosinophils, monocytes and basophils. Notably, co-administration of MelB and GB was significantly superior to T.b.r-induced in stabilizing neutrophil levels. This is a significant observation given that severe inflammation is an important hallmark for T.b.r-driven pathology in the brain. T.b.r-driven eosinophilia was also observed which was blocked by administration of GB and MelB; perhaps attributable to the anti-inflammatory properties of GB. Notably, T.b.r-induced leukocytopenia has been reported in various studies [47–48]. In the current study, leukocytopenia was observed in infected mice, accompanied by neutrotopenia, which could predispose the infected animals to other infections [47].

In the present study, a T.b.r-induced decrease in the levels of lymphocytes shows trypanosome-driven suppression of lymphoproliferative responses due to the increased burden of T.b.r infection. This outcome is consistent with a prior study on T.b.r infection [49–50]. Notably, GB significantly restored T.b.r-induced changes to lymphocytes. This clearly demonstrates GB-driven immune modulation—the mechanism by which GB attenuates T.b.r-driven alteration of lymphocytes could be multifactorial possibly due to its antioxidant and immune-stimulatory properties [51].

The thrombocytopenia observed during T.b.r infection could be attributed to damage to platelets by ROS [52–53]. Low platelet counts could also be attributable to the pooling of blood in the spleen, removal of platelets by the mononuclear phagocytic system and increased ‘consumption’ of platelets by disseminated intravascular coagulation reaction during trypanosomiasis infections [54–55]. The decline in platelet count was notably blocked when MelB or GB were administered separately or in combination. However, there was no significant difference between T.b.r+MelB and T.b.r+MelB+GB in regard to platelet levels. Notably, GB is known to inhibit platelet aggregation, both in platelet-rich plasma (PRP) and whole blood; and can therefore can be used as an oral anti-platelet therapeutic agent [56]. It is likely that the free radical scavenging power of GB played a role in ameliorating T.b.r-driven thrombocytopenia. As alluded to by Gohil and Packer [37], EGb 761 is capable of scavenging hydroxyl radicals since it possesses superoxide dismutase-like activity. In light of the current findings and prior studies on the potential role of GB on platelets, this phenomenon requires further scrutiny to unravel clear mechanism involved. Obviously, intake of GB may have important implications for patients at risk of deep vein thrombosis as well as those on blood thinning medications.

The host metabolism is usually affected by trypanosomes in the adipose tissue. T.b.r infection led to the elevation of cholesterol and triglycerides and decline in HDL levels. The hydrolysis of triglycerides to free fatty acids via beta-oxidation is believed to be due to the ability of trypanosomes to utilize beta fatty oxidation for their energy needs [57]. Other studies have demonstrated that *Trypanosoma cruzi* parasite depletes HDL, which in turn results in excess cholesterol transfer from the periphery to the liver. High levels of triglycerides and low HDL level disrupts mitochondrial function, resulting in increased ROS, flawed endothelial system and inflammation [58]. A previous study demonstrated that GB modulates the metabolism of cholesterol. A study by Rodriguez et al. [59] showed that GB has the capacity to lessen the emergence and size of atherosclerotic plaques in cardiovascular patients who are at high risk, reduce lipid peroxidation, and diminish lipoprotein levels. This observation is consistent with an earlier study that demonstrated the ability of *Ginkgo biloba* to reduce serum total cholesterol and triglycerides [56]. Other notable studies showed that GB has an effect on the absorption of triglyceride and cholesterol as well as cholesterol metabolism; a possible explanation for the ability of *Ginkgo biloba* to lower the level of lipids, and therefore deter the development and progression of artherosclerosis and cardiovascular diseases [60–62]. These prior studies corroborate findings from the current study that show MelB and GB-driven abrogation of T.b.r-induced elevation of cholesterol and triglycerides as well as depletion of HDL.

At this point, it is important to note that dyslipidemia is usually associated with the induction of oxidative stress, by free radicals. In both the early and late stages of HAT, free radicals are produced resulting in cellular injury. The free radicals bombard membrane lipids as well as proteins, ultimately overwhelming the endogenic antioxidant capacity driven by reduced glutathione (GSH), superoxide dismutase, and catalase [63]. This study demonstrated that T.b.r infection resulted in the depletion of brain and liver GSH, a characteristic indicator for oxidative stress. Depletion of GSH is attributed to overwhelming oxidative stress that decimates the antioxidant system. Moreover, mice infected with T.b.r showed an increase in GSH levels in the spleen, heart, kidney and lungs. Such an increase signifies an antioxidant response to rising oxidative stress. The rise in GSH levels is typical feedback to rising oxidative stress. Overwhelming levels of oxidative stress and severe inflammation are usually present during the meningoencephalitic stage of HAT [64]. Furthermore, it is worth noting that melarsoprol as well as its metabolite, melarsen oxide generates reactive oxygen species (ROS) and leads to oxidative stress [6,13]. The results from this study are consistent with those of Li et al. [65] and Kaur et al. [66], in which GSH was elevated in neuronal cells when GB was administered due to its antioxidant activity. In the current study, it was prudent to determine of co-administration of MelB and GB assuaged MelB-induced toxicity. Indeed, comparisons between T.b.r+MelB+GB and MelB+T.b.r show that co-administration was more effective in stabilizing GSH levels for the brain. The presence of GB had a modulatory effect on GSH levels in all the organs (brain, spleen, heart, kidney, lungs and liver) in the presence of T.b.r and MelB. The possible role of GB in protecting from T.b.r and MelB-induced negative physiological and biochemical effects during late stage HAT has potential for improving treatment outcome, and therefore requires further scrutiny through dose-response studies.

Trypanosome invasion is usually associated with elevation of free radicals such as nitric oxide [67–68] and depletion of systemic antioxidants. In a study by Eze et al. [69], elevation in the serum and organ lipid peroxidation during *Trypanosoma brucei* infection in rats was reported. In the current study, T.b.r infection and treatment with MelB led to an increase in MDA relative to the uninfected control group, signifying a rise in lipid peroxidation. Brain, liver, kidney and serum MDA were significantly elevated (p<0.001, Fig 7E) in T.b.r infected mice when compared to the control. It is also notable that MelB administration resulted in significant elevation of MDA in the brain, liver and kidney (Fig 7A, 7B and 7D). Elevation of MDA is an established characteristic indicator of active lipid peroxidation. Notably, co-administration of GB and MelB (T.b.r+MelB+GB) significantly attenuated T.b.r and MelB driven elevation of MDA for the liver and serum (Fig 7B and 7E). MDA levels for the brain and kidney demonstrate superior protection from GB, when compared to MelB. These results corroborate studies in which EGb 761 reduced MDA levels and effectively reduced cataractogenesis in Sprague-Dawley rats and Wistar rats [70–72]. Another study found that *Ginkgo biloba* reduced MDA and protected against silver nanoparticles-induced neuronal apoptosis and blood-brain barrier impairments in rats [73]. Furthermore, other studies have demonstrated the ability of GB to reduce lipid peroxidation and protect neuronal cells against acute hypoxia and improve cognitive functions through the improvement of neuronal lesions in Sprague-Dawley rats [65–66].

The depletion of tissue antioxidant systems has been linked to membrane oxidative stress and cellular destruction during trypanosomiasis [74]. A study done on ervet monkey species infected with *Trypanosoma brucei rhodensiense* showed elevated nitric oxide in serum samples [68]. This is clearly supported by the current study, in which T.b.r infection as well as treatment with MelB elicited high nitrite levels in mice serum. However, administration of GB significantly stabilised levels of nitrite in infected mice and those infected (treated with MelB); signifying a robust protective effect against T.b.r and MelB-driven elevation of nitrite. These findings are consistent with those of Lee et al. [75], which concluded that the expression of pro-inflammatory mediators, such as TNF-α, IL-6, macrophage inflammatory protein (MIP)-2, nitric oxide synthase (NOS) and COX-2, was suppressed in adult mice by GB.

Serious inflammatory reactions are triggered during trypanosomiasis. These responses are mediated by the discharge of pro-inflammatory cytokines such as interferon-gamma (INF-γ), tumor necrosis factor (TNF-α), IL-1, IL-2 and IL-6 [76]. Uncontrollable pro-inflammatory cytokines have been reported as a result of inflammation and anaemia caused by trypanosomes [43]. As per the current study, T.b.r infection led to heightened levels of pro-inflammatory cytokines IFN-γ and TNF-α. However, there was no significant change in IL-10 levels in infected mice. INF-γ is responsible for the adaptive immune reaction in the course of T.b.r infection. In a study by Hertz & Mansfed [77], increased INF-γ led to a decline in parasite count. Additionally, in a different study, IFN-γ -knockout mice resulted in uncontrollable parasitaemia levels as well as decreased survival rates [78].

The parasites usually shed variant surface glycoproteins which trigger the production of TNF-α. In addition, macrophages emit this pro-inflammatory cytokine which is essential for cell growth, differentiation as well as apoptosis. In the brain, the rising level of TNF-α aids the trypanosome to cross the blood-brain barrier leading to acute neuropathy [79]. This important finding may explain the effect of the parasites on the blood-brain barrier. The increased levels of both TNF-α and IFN-γ may, perhaps play a role in the breach of the blood-brain barrier by the parasites. GB can impede the sequel of inflammatory agents like lipopolysaccharide (LPS) by enhancing the activity of transforming growth factor (TGF), which leads to a decline in the gene expression of Interleukin-1 (IL-1), IL-6, and tumor necrosis factor alpha (TNF-α), ultimately decreasing inflammatory processes. Notably, the T.b.r-driven elevation of TNF-α was diminished by pre-treatment with GB, a finding consistent with earlier studies demonstrating the role of GB extract in anti-inflammatory processes [80]. The infected mice showed a remarkable increase in serum TNF-α as well as INF-γ relative to those in the control group; an indication of inflammation. Notably, both treatment (MelB and GB) and pre-treatment with GB reduced of pro-inflammatory cytokine IFN-γ and TNF-α. Additionally, the elevation of the pro-inflammatory cytokines TNF-α and IFN-γ was blocked (p<0.001) by co-administration of GB and MelB; when compared with mice infected with T.b.r or treated with MelB. These observations are consistent with ameliorative effects noted in mice administered with GB. This finding may also explain the molecular basis for the ability of GB to protect the blood-brain barrier integrity, since the elevation of TNF-α drives breach of the blood-brain barrier in T.b.r infection. Moreover, ginkgolide A, a component of *Ginkgo biloba*, has the capacity to counter cyclo-oxygenase-2 (COX-2) and 5-lipo-oxygenase (5-LOX), which are limiting enzymes involved in the conversion of arachidonic acid to prostaglandin and leukotrienes respectively, as well as reduction of endoplasmic reticulum stress, which contributes to inflammation [81]. Moreover, neutrophils, eosinophils, monocytes and T-lymphocytes share a multifaceted association and; collectively, they coordinate a more heightened immune response by regulating the secretion of pro-inflammatory cytokines against Trypanosome parasites; nonetheless, this association must be strongly controlled as it may contribute to explicit inflammation and inception of pathology. There is a need for further studies on how GB modulates pro-inflammatory cytokines in late-stage HAT. This may open new possibilities for controlling inflammation during HAT.

At the onset of trypanosomiasis, IL-10 downregulates IFN-γ and TNF-α secretion relieving inflammatory feedback [43]. After long-term exposure to the parasite, ROS increase and elevate pro-inflammatory cytokine action which overwhelms the activity of the anti-inflammatory cytokine IL-10 [79]. There was no significant change in IL-10 levels after infection with T.b.r or treatment with melarsoprol and EGb 761.

The kidney and the liver are vital organs usually damaged by the unstable radicals produced during trypanosomiasis [63]. Elevated levels of ALT, AST and ALP were noted in infected mice. The heightened levels of these biomarkers signified active liver damage. A comparative analysis of MelB or GB treatment to T.b.r infected mice and the normal control, revealed a significant normalization of the serum levels of ALT (p<0.001) and AST on exposure to MelB or GB. Additionally, there was a significant abrogation of T.b.r-driven elevation of AST in infected mice co-administered with GB and MelB (T.b.r+MelB+GB) in comparison to MelB (T.b.r+MelB) treated mice. Notably, MelB when administered alone, was most effective in stabilizing AST levels in the presence of T.b.r. Only GB was able to significantly attenuate T.b.r-driven elevation of AP. However, there was no statistical difference in GGT levels across all experimental groups. Administration of MelB and GB improved the T.b.r-driven hepatotoxicity, as demonstrated by low levels of ALT, AST and ALP, consistent with previous studies [82–83]. The role of MelB in killing T.b.r parasites may play an important role in its ability to stabilize liver enzymes. The desirable hepatoprotective impact of GB may be ascribed to its ability to regulate endogenous antioxidant mechanisms, which incredibly modulate liver toxicity in various experimental models [80].

In the current study, we observed an elevation of urea and uric acid in T.b.r infected mice. Notably, albumin levels decreased in infected mice in line with previous study by Anosa, [84]. MelB was the most effective in countering T.b.r-driven rise in urea and uric acid when compared to GB. This finding may suggest that changes to urea and uric acid are driven parasites; that are killed in the presence of adequate concentrations of MelB. Significantly low albumin levels were observed in infected mice, when compared to those in the normal control group; a phenomenon that was blocked when GB was given together with MelB. This observation corroborates previous studies [85]. However, no statistical difference was observed in creatinine levels across all experimental groups.

T.b.r infection resulted in a generalised decrease in serum levels of sodium, potassium and chloride ions signifying metabolic acidosis, in contrast to prior findings by Karaye et al. [86] in *Trypanosoma congolense* and *Trypanosoma brucei* infected red Sokoto bucks. However, the findings are consistent with those of Ojo et al. [87] in *Trypanosoma brucei brucei* infected rats. Both MelB and GB when administered separately or when co-administered, significantly normalized K, Na and Cl levels in serum. The role of MelB may be linked to destruction of parasites whereas, for GB, the effect may be attributed to its role in the protection of the membrane ultrastructure against free radicals, modulation of some enzymatic systems and ionic pumps [88].

## Conclusion

In conclusion, findings from the current study demonstrate that administration of *Ginkgo biloba* ameliorate HAT pathogenesis by reducing oxidative stress, inflammation and organ damage caused by T.b.r infection as well as toxicities due to MelB. The findings provide a basis for further studies to elucidate the potential use of *Ginkgo biloba* as an adjuvant therapy, that may improve treatment outcomes when used alongside melarsoprol for severe late stage of HAT. Of particular interest will be dose response studies to establish optimal formulations and dosage with maximal benefits, as well as identification of specific chemical components in GB that confer its beneficial effects.
